# On-Pump Bidirectional Glenn Shunt Compared to Off-Pump Shunt Is a Better Influencing Determinant of Perioperative Morbidity During Palliation of Univentricular Heart: Compilation of Two Cases

**DOI:** 10.7759/cureus.28343

**Published:** 2022-08-24

**Authors:** Vishal V Bhende, Hardil P Majmudar, Tanishq S Sharma, Ashwin S Sharma, Amit Kumar, Rahul Tandon, Purvi R Patel, Nirja Patel, Gurpreet Panesar, Kartik B Dhami, Kunal Soni, Sohilkhan R Pathan

**Affiliations:** 1 Pediatric Cardiac Surgery, Bhanubhai and Madhuben Patel Cardiac Centre, Bhaikaka University, Karamsad, IND; 2 Physiology, Gujarat Cancer Society Medical College, Hospital and Research Centre, Ahmedabad, IND; 3 Pediatric Cardiac Intensive Care, Bhanubhai and Madhuben Patel Cardiac Centre, Bhaikaka University, Karamsad, IND; 4 Pediatrics, Pramukhswami Medical College & Shree Krishna Hospital, Bhaikaka University, Karamsad, IND; 5 Cardiac Anaesthesiology, Bhanubhai and Madhuben Patel Cardiac Centre, Bhaikaka University, Karamsad, IND; 6 Clinical Research Services (CRS), Bhanubhai and Madhuben Patel Cardiac Centre, Bhaikaka University, Karamsad, IND

**Keywords:** off-pump glenn shunt, cardiopulmonary bypass., univentricular palliation, cavopulmonary shunt, on-pump bi-directional glenn, bi-directional glenn shunt

## Abstract

The bidirectional Glenn (BDG) or hemi-Fontan technique, performed under cardiopulmonary bypass (CPB) and often utilized as first-stage palliation for various cyanotic congenital heart diseases, as a part of the single-ventricle repair is associated with adverse side effects and high expenditures. Previous studies have shown that BDG is safe even without CPB, which thus necessitates further investigation. This manuscript presents the case of two patients with complex cyanotic congenital heart diseases. The first case was an 11-month-old baby who presented with fever, cough, and cold, while the second case was a two-year-old baby who was underweight due to poor feeding status. Both patients underwent a BDG and main pulmonary artery partial ligation following the requisite preoperative medical evaluations on CPB. Moreover, case 1 had atrial septectomy, while case 2 had significant aortopulmonary collateral arteries ligation. Both patients were discharged following an uneventful postoperative outcome. We found that an adequate selection of patients for the BDG procedure, with or without CPB, could lead to identical postoperative outcomes with regard to mortality, morbidity, and supply usage.

## Introduction

One of the most common surgical techniques for cyanotic congenital heart diseases (CHD) is the bidirectional cavopulmonary (Glenn) shunt, which is frequently performed in palliation of single-ventricle repair [1]. It accounts for one-half of ventricular repairs in hypoplastic right ventricle cases and Ebstein’s anomaly. The surgery is performed to alleviate volume overload. This is usually done under cardiopulmonary bypass (CPB), which is accompanied by risks and expenditures. The CPB has also been linked to a significant increase in proximal superior vena cava (SVC) pressure during this surgical procedure, which could result in neurological complications. Conversely, some authors have attested to the safe health outcome of bidirectional Glenn (BDG) without CPB [2-9], which must be further investigated to suggest an optimal procedure in clinical practice. This manuscript discusses the perioperative health outcomes for two cases of complex cyanotic CHD that underwent on-pump BDG shunt.

## Case presentation

The Institutional Ethics Committee (IEC-2) at the HM Patel Centre for Medical Care and Education in Anand, Gujarat, approved this study (Approval No. IEC/BU/2022/Cr.02/163/2022 dated 30.07.2022), and the formal informed consent was obtained from the patient's parents before surgery.

Case 1

In the pediatric cardiac outpatient department, a male baby of 11 months with a 7.2 kg weight was presented by his parents with serious complaints of once to twice-monthly episodes of fever, cough, and cold. He also had a history of poor weight gain and an inability to establish adequate complementary feeding. He exhibited bluish darkening of the nail bed and lips, as well as a propensity for crying excessively. The following clinical information was discovered in his birth medical record: the baby was born via lower-segment cesarean section with meconium aspiration. He had previously spent seven days in the neonatal intensive care unit with oxygen support. As of nine months of age, the patient has received all his requisite vaccines.

The patient had complex cyanotic CHD, single-ventricle physiology with tricuspid valve atresia (Type 1B), restrictive atrial septal defect, shunting right to left, and a minor ventricular septal defect, as revealed by a two-dimensional (2D) echocardiography examination. The left ventricle was dominating, while the right ventricle was hypoplastic. The right atrium (RA) was serviced by a single SVC. The patient also exhibited situs solitus, levocardia, and preoperative room air saturations of 65%-75%.

Prior to the surgery, the patient underwent a thorough preoperative clinical assessment, including cardiac and neurological evaluation. In addition, regular laboratory investigations were undertaken using qualitative molecular analysis to screen the patient for SARS-CoV-2 (COVID-19), which was negative. Following evaluation and preparation, he was administered midazolam (0.5 mg/kg) before being placed under general anesthesia. Capnography for end-tidal carbon dioxide was one of the parameters used for routine intra-operative monitoring. The monitoring also included a five-lead electrocardiogram and pulse oximetry. After his intubation, the following items were secured in place: an intra-arterial line and a central venous line in the internal jugular vein (to estimate the SVC pressure during clamping to evaluate the BDG adequacy). However, a triple lumen catheter was also placed in the femoral vein. The ventilation system was adjusted to maintain steady levels of partial pressure of carbon dioxide and oxygen in arterial blood (paCO_2_ and paO_2_), respectively. After intubation, the child was put on the pressure control mode of ventilation keeping target etCO_2_ in the range of 40-45 mm Hg.; FiO_2_ was titrated to adjust on-screen saturation between 80% and 85%.

**Figure 1:**
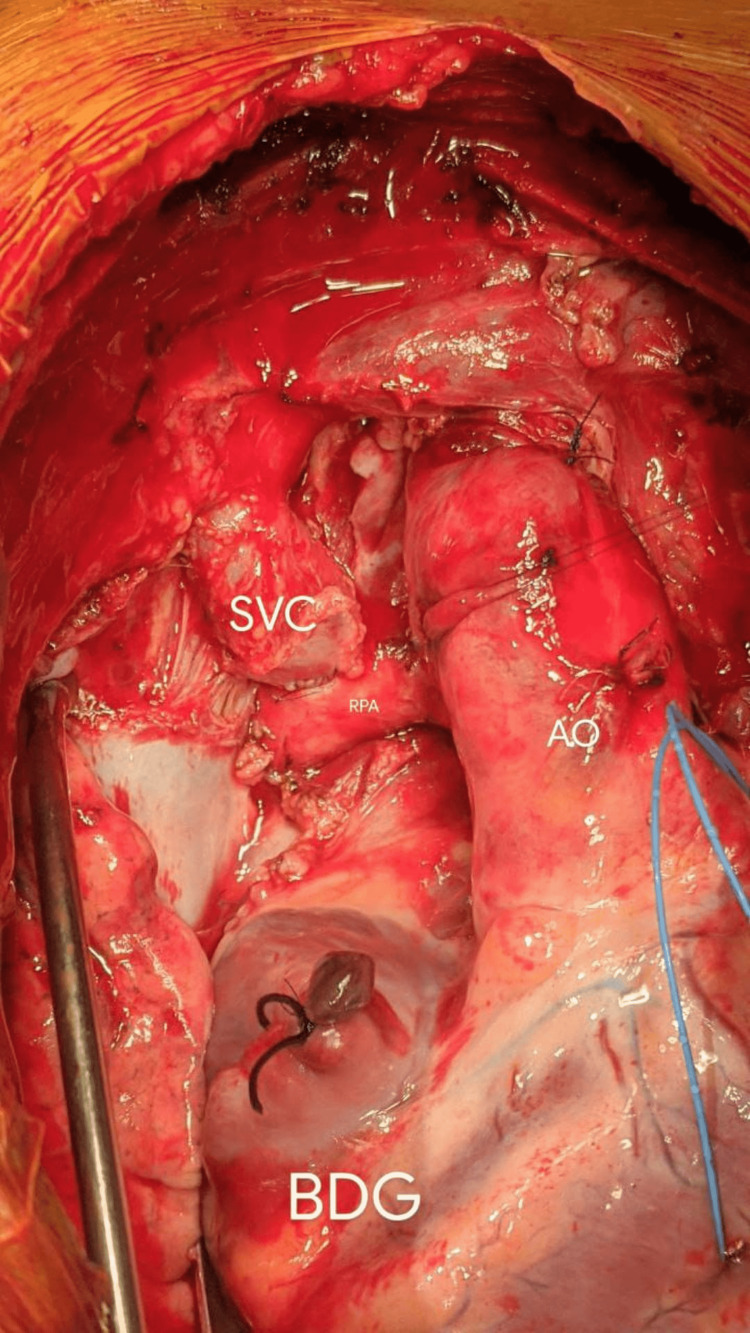
Completed end-to-side anastomosis of superior vena cava with right pulmonary artery (bidirectional Glenn shunt) BDG: bidirectional Glenn shunt; SVC: superior vena cava; RPA: right pulmonary artery; AO: aorta. Image credits: Dr. Vishal V. Bhende.

During the surgery, we performed a standard median sternotomy and opened the pericardium. Later we dissected the SVC and ligated the azygos vein from the right atrial end to the innominate vein junction. On the medial and lateral portions of the SVC, marking sutures were placed, to correspond to the right pulmonary artery (RPA) arteriotomy. That will in turn provide correct positioning during the BDG and avoid anastomosis distortion. From the bifurcation to the hilar region area, we dissected RPA. Then we separated SVC from the RA and closed the atrial end with 6/0 or 5/0 polypropylene continuous sutures. End-to-side anastomosis between the divided SVC and incised RPA was made after performing a wide incision in the dissected RPA. However, the used suture was 6/0 or 7/0 polypropylene (Figure 1).

**Figure 2:**
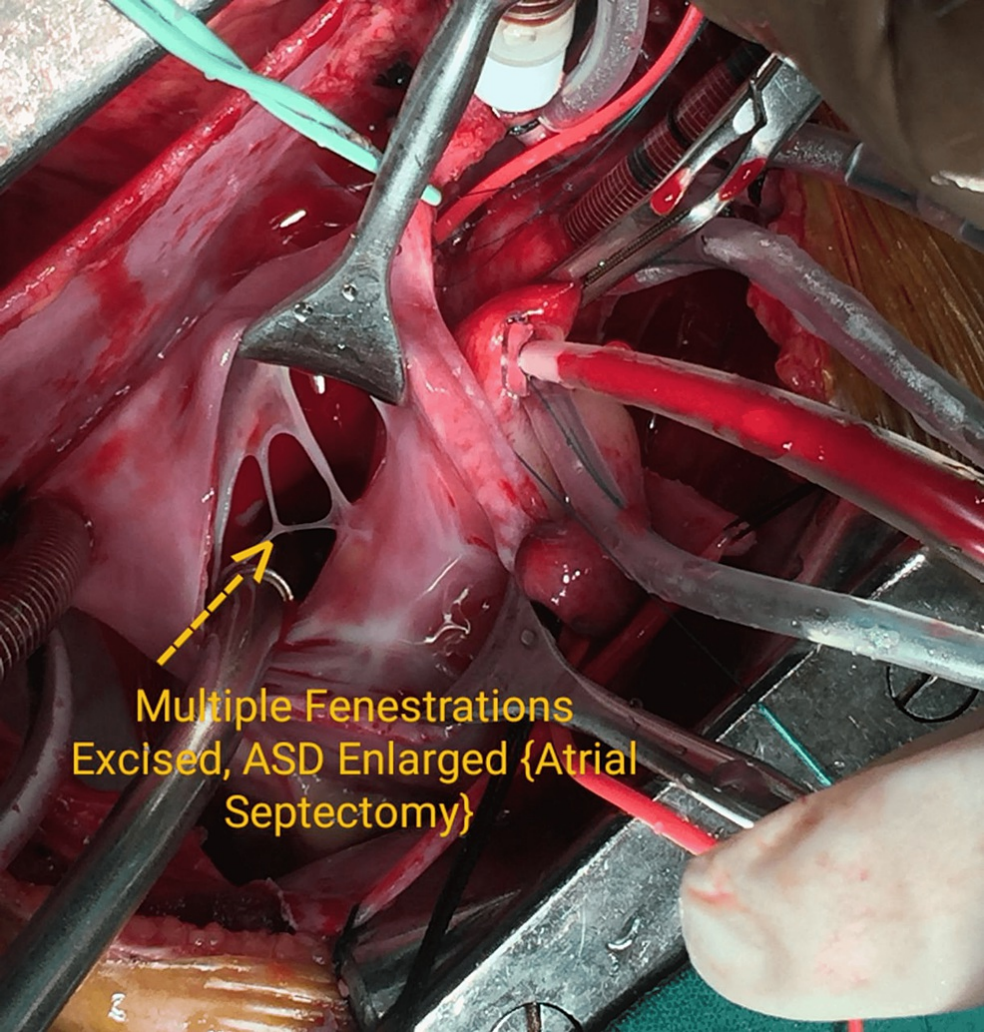
Atrial septectomy (surgical) procedure ASD: atrial septal defect. Image credits: Dr. Vishal V. Bhende.

**Figure 3:**
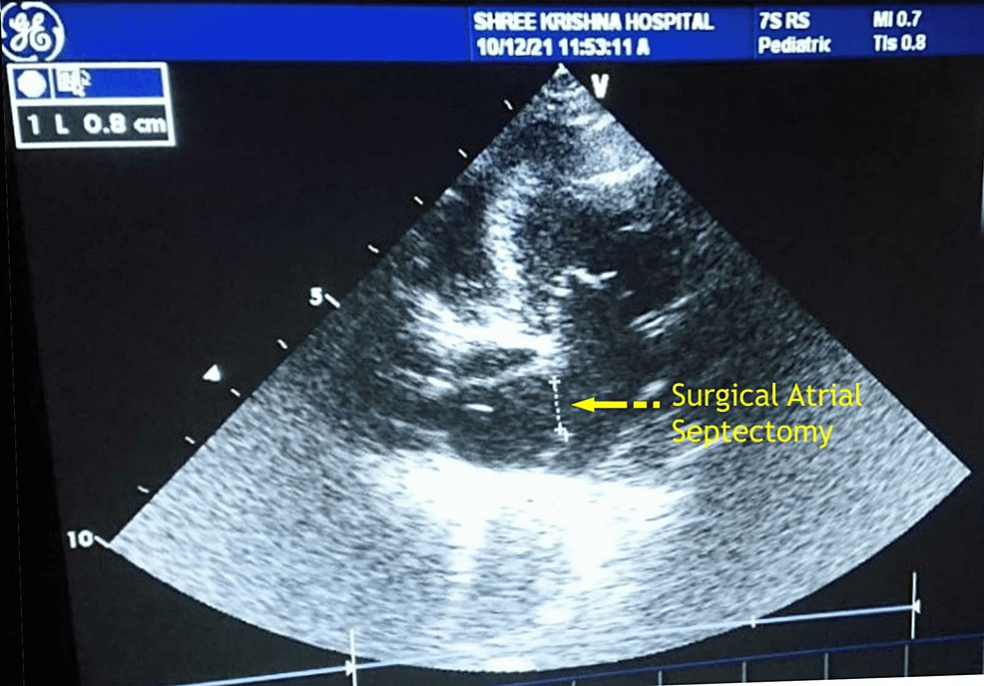
Confirmation of the atrial septectomy procedure with the 2D echocardiography in case 1. 2D: two-dimensional. Image credits: Dr. Vishal V. Bhende.

As the patient’s 2D echocardiography revealed a restricted atrial septal defect, atrial septectomy was performed in addition to the BDG operation (Figures 2, 3).

Following the surgery, the patient had smooth and uneventful postoperative outcomes. He maintained a room air saturation of 85% at the time of discharge. Details of the outcomes are presented in Table 1.

**Table 1:** Intra- and postoperative parameters of both cases CPB: cardiopulmonary bypass; ACC: aortic cross-clamp; BDG: bidirectional Glenn shunt; MPA: main pulmonary artery; MAPCAs: major aortopulmonary collateral arteries; PA: pulmonary artery; SVC: superior vena cava; IVC: inferior vena cava; CVP: central venous pressure.

	Case 1	Case 2
Age	11 months	2 years
Sex	Male	Male
Weight	7.2 kg	7.8 kg
CPB duration	83 min	68 min
ACC duration	60 min	-
Time on ventilator	11 hours	26 hours (1 day)
ICU stay	5 days	5 days
Hospital stay	13 days	10 days
Surgical procedure performed + associated procedures	BDG; atrial septectomy; MPA partial ligation (PA banding)	BDG; MPA partial ligation (PA banding); MAPCAs ligation
2D Echocardiography during discharge time	There was a right-sided bidirectional Glenn shunt, well flowing, with no obstructions. Moderate atrial septal defect, shunting right to left	Well-flowing right-sided bidirectional Glenn shunt. There was no obstruction in the Glenn. There was mild narrowing at the right pulmonary artery origin. There was no significant gradient
On discharge, SVC/Glenn pressure; IVC/CVP	29/22 (25) mmHg (Glenn); 7 mmHg	20/15 (17) mmHg (Glenn); 7/4 (5) mmHg

Case 2

On the third day of life, a two-year-old toddler weighing 7.8 kg was hospitalized due to difficulty in feeding. Following clinical investigations, he was diagnosed with sepsis, linked to congenital heart disease. The treatments administered were as follows: intravenous fluids, antibiotics, phototherapy, and supportive therapy. The baby's medical record stated that the baby was a full-term male baby with spontaneous vaginal delivery and birth weight of 2.65 kg with immediate crying after birth.

The 2D echocardiography procedure was performed with similar outcomes as obtained in case 1. In addition, this baby underwent a cardiac computed tomography (CT), which revealed confluent branch pulmonary arteries (PAs) as follows:

-RPA: proximal, 5.8 mm; distal, 5.6 mm

-Left pulmonary artery: proximal, 7.8 mm; distal, 7.2 mm

-Main pulmonary artery (MPA): proximal, 12.1 mm; distal, 11.5 mm

-Mc Goon, 1.153; Nakata Index, 105.42 mm^2^/m^2^.

A complete preoperative evaluation, including cardiac and neurological assessments, was performed including all the laboratory investigations as indicated previously in case 1. We also confirmed the negativity of the qualitative molecular analysis for SARS-CoV-2 (COVID-19). Subsequently, he was orally pre-medicated with midazolam and then placed under general anesthesia. All the routine intra-operative monitoring procedures for intubation were similarly performed as in case 1. The invasive lines were placed on the patient, while the ventilation was adjusted to maintain adequate paCO_2_ and paO_2_. The surgical procedure included BDG shunt, MPA partial ligation, and major aortopulmonary collateral arteries ligation.

The patient also had a smooth and uneventful postoperative outcome similar to case 1.

**Figure 4:**
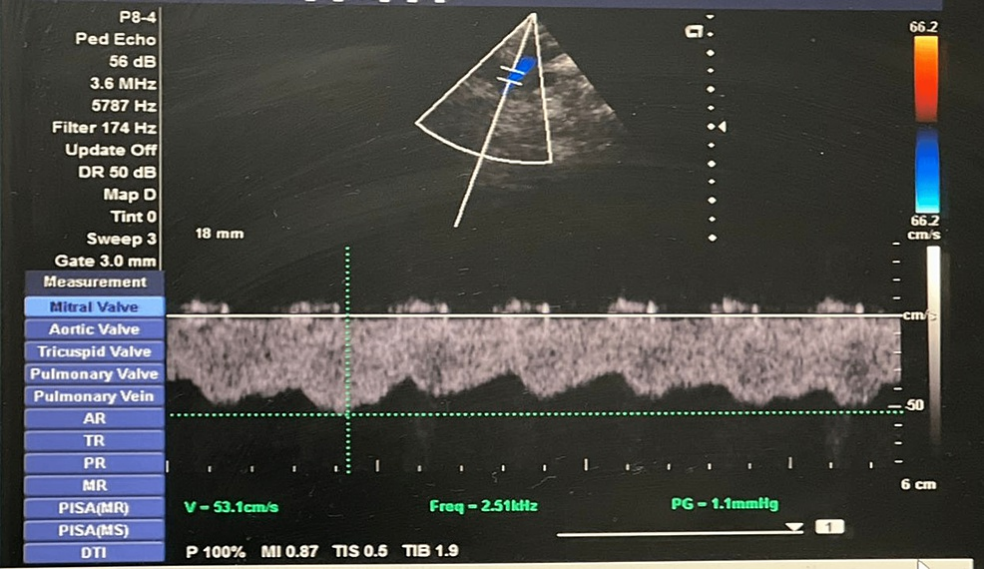
Doppler interrogation of the bidirectional Glenn shunt from the suprasternal window showing phasic low-velocity laminar flow Image credits: Dr. Vishal V. Bhende.

The 2D echocardiography was performed in the postoperative period before discharge (Figure 4).

The Glenn flow was kept at the laminar phase with accentuation during atrial systole and ventricular diastole while maintaining a velocity of 0.5 m/s (Table 2).

**Table 2:** Beneficial effects of bidirectional Glenn shunt on pump (CPB) CPB: cardiopulmonary bypass; SVC: superior vena cava; LPA: Left pulmonary artery; MUF: modified ultrafiltration; BDG: bidirectional Glenn; INR: Indian rupee; USD: United States dollar.

Sr. No.	Description
1.	Mild hypothermia achieved through CPB leads to cerebral protection.
2.	When the SVC was clamped with CPB, minor reductions in the diastolic, mean, and peak systolic blood flow velocities of the middle cerebral artery were observed compared to major reductions (75%) when the SVC was clamped without CPB.
3.	Associated procedures required for the patient can be performed as follows: Atrial septectomy: Enlarging the atrial septal defect, done by opening the right atrium. LPA plasty if LPA has proximal stenosis done by augmenting it with a treated/untreated pericardial patch.
4.	Post-BDG anastomosis, CPB, and performing MUF reduce the increased pulmonary vascular resistance and fluid sequestration and improve myocardial function.
5.	High hematocrit in cyanotic patients gets corrected on-pump to normative hematocrits as found in the general population, which in turn improves oxygenation for the patient.
6.	Anastomosis of BDG can be performed leisurely on-pump, and having a tension-free anastomosis is essential for the performance of the shunt.
7.	As an added benefit of performing these treatments on the pump (according to Indian scheme/package laws), for Glenn procedure on CPB and without CPB the execution cost is Rs. 1 lac INR (1260.19 USD). When the extracorporeal circuit is not needed, the surgery can be completed with significant equipment cost savings.
8.	Pristine surgical field
9.	Uniform anastomosis
10.	Avoidance of neurological injury during SVC clamping

## Discussion

The three main problems of off-pump BDG shunt are as follows: a) difficulty in performing the surgery quickly so as to curtail the time spent clamping the SVC; b) the long-duration consequences on the brain due to high SVC pressures; and c) the necessity to decompress the SVC with temporary shunts. The traditional Glenn shunt was performed without CPB through a thoracotomy. Glenn had partially blocked the SVC in his original report. However, in clinical practice, many shunts have been performed focusing on complete SVC occlusion and also do not have any neurological deficit. Lamberti et al. [2] illustrated the conduct of BDG in seven patients who did not have CPB. One of those patients had no intra-operative shunt but had bilateral SVC. In the rest of the six cases, intra-operative SVC-right atrial temporary shunt was employed with the usage of standard vena cava cannulas for shunting, taking precautions to prevent air embolism.

**Figure 5:**
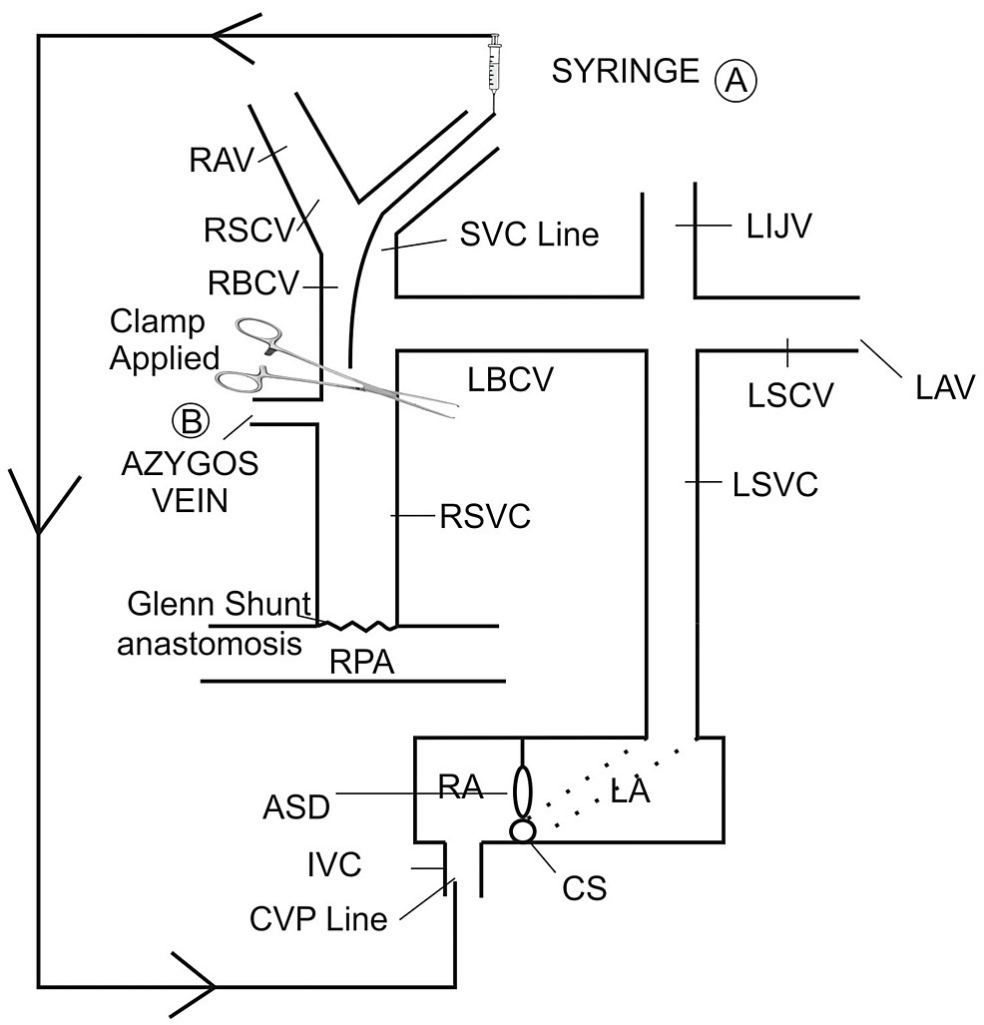
Diagrammatic representation of methods of decompression of SVC during off-pump BDG A: active decompression; B: passive decompression; RAV: right axillary vein; RSCV: right subclavian vein; RBCV: right brachio-cephalic vein; LBCV: left brachio-cephalic vein; LIJV: left internal jugular vein; LSCV: left subclavian vein; LAV: left axillary vein; LSVC: left superior vena cava; RSVC: right superior vena cava; RA: right atrium; LA: left atrium; ASD: atrial septal defect; CS: coronary sinus; IVC: inferior vena cava; CVP: central venous pressure. Image credits: Dr. Vishal V. Bhende.

Bilateral SVC precludes the use of a temporary shunt. Decompression of the SVC has been described using temporary shunts, which were either placed in between the RA and the SVC or in the contralateral PA. As a result, the azygos vein that was left open during anastomosis formation is an internal shunt [10]. Other schools of thought suggest that it is illogical to have the involvement of the valved azygos vein in decompressing the SVC. The SVC can be passively softened, by elevating the venous pressure that drives the blood flow between cannulas in the RA and the SVC or PA. Alternatively, insert a roller or centrifugal pump that entails active decompression [11] into the circuit to drain the SVC. A prior paper recommended using a syringe to aspirate the SVC, which would lead to decompression [12] (Figure 5).

Although using a shunt appears to be plausible [10], our study and that conducted by Jahangiri [7] categorize its usage as inconsequential. Jahangiri et al. developed the idea of brain CT scan and the developmental assessment of the patients before and after the BDG shunt off-pump surgery. However, one study reported seven cases with BDG shunt and no CPB or shunting which had their SVC clamped [7]. The SVC pressure range was 19-65 mm Hg (median = 26 mm Hg) during the clamping, while they kept the transcranial pressure (TCP) (i.e., cerebral perfusion) at 30 mm Hg at all times. This was achieved by employing inotropic drugs as needed without any associated neurological injury. These conclusions are not supported by the head CT scan or psychometric/developmental testing which is performed to detect any faint brain injury. TCP or cerebral perfusion is actually the difference between the systolic arterial pressure and the mean jugular venous pressure. During clamping, the value of SVC pressure can rise as high as 60 mm Hg; thus, inotropes are strongly recommended to boost the mean arterial pressure. It is expected that by keeping the TCP above 30 mm Hg, adequate cerebral perfusion will be maintained. The TCP (cerebral perfusion) is defined as systolic arterial pressure minus mean jugular venous pressure or mean arterial pressure minus mean SVC pressure, both of which must be ≤30 mm Hg.

Multiple neurodevelopmental measures created by various authors are available to assess cognitive outcomes following heart surgery. In this manuscript, Gesell’s developmental [13] and Vineland’s social maturity scale were implemented to check the efficacy of modified surgical strategy in patients having the Glenn shunt surgery [14].

A modified motor and mental developmental test for Indian babies [15] was also employed (Table 3).

**Table 3:** Criteria for performing BDG shunt. Trans-pulmonary gradient is the difference between the central venous pressure and left atrial pressure; it determines the pulmonary blood flow. BDG: bidirectional Glenn.

Sr.No.	Description
1.	Currently, the accepted optimal age for the BDG is 3-9 months
2.	The pulmonary artery mean pressure should be less than 18 mm Hg. Alternatively, 15 mm Hg is ideal
3.	Calculated pulmonary vascular resistance is less than 2 units/m^2^
4.	In terms of postoperative hemodynamics, the estimated safe pulmonary artery index should be greater than 250 mm^2^/m^2^
5.	Mild atrioventricular regurgitation
6.	The mean ventricular end-diastolic pressure is less than 12 mm Hg
7.	The accepted trans-pulmonary gradient ≈5-8 mm Hg

## Conclusions

Even though cavopulmonary anastomosis is a short shunt, the patient’s age, PA size, pressure measurement, ventricular assessment, and other concomitant defects are the determinants of the clinical outcome. The BDG surgical procedure can be performed with or without CPB support with no substantial changes in operative mortality, morbidity, or resource utilization. Therefore, an off-pump BDG procedure should be considered a safe alternative to the traditional use of CPB for an adequately selected group of patients. Otherwise, the benefits of on-pump BDG, as outlined in the preceding section, are a superior option for stepwise univentricular route mitigation.
